# Platelet derived growth factor receptor alpha mediates nodal metastases in papillary thyroid cancer by driving the epithelial-mesenchymal transition

**DOI:** 10.18632/oncotarget.13299

**Published:** 2016-11-11

**Authors:** Esther Ekpe-Adewuyi, Ana Lopez-Campistrous, Xiaoyun Tang, David N. Brindley, Todd P. W. McMullen

**Affiliations:** ^1^ Department of Surgery, University of Alberta, Edmonton, Canada; ^2^ Signal Transduction Research Group, Department of Biochemistry, University of Alberta, Edmonton, Canada; ^3^ Division of Surgical Oncology, Cross Cancer Institute, University of Alberta, Edmonton, Canada

**Keywords:** papillary thyroid cancer (PTC), platelet derived growth factor receptor-alpha (PDGFRα), epithelial to mesenchymal transition (EMT), invadopodia, crenolanib

## Abstract

Recently platelet derived growth factor receptor-alpha (PDGFRα) was recognized as a potential target to treat aggressive papillary thyroid cancer given its strong association with lymph node metastases. However, it is unclear how PDGFRα potentiates metastases and if it works through the canonical MAPK pathway traditionally linked to PTC oncogenesis. We explored the phenotypic changes driven by PDGFRα activation in human papillary thyroid cancer (PTC) cells and the downstream signalling cascades through which they are effected. We demonstrate that PDGFRα drives an impressive phenotypic change in PTC cell lines as documented by significant cytoskeletal rearrangement, increased migratory potential, and the formation of invadopodia. Cells lacking PDGFRα formed compact and dense spheroids, whereas cells expressing active PDGFRα exhibited invadopodia in three-dimensional culture. To achieve this, active PDGFRα provoked downstream activation of the MAPK/Erk, PI3K/Akt and STAT3 pathways. We further confirmed the role of PDGFRα as a transformative agent promoting the epithelial to mesenchymal transition of PTC cells, through the augmentation of Snail and Slug expression. Crenolanib, a small molecule inhibitor of PDGFRα, suppressed the levels of Snail and Slug and almost completely reversed all the phenotypic changes. We demonstrate that PDGFRα activation is an essential component that drives aggressiveness in PTC cells, and that the signaling pathways are complex, involving not only the MAPK/Erk but also the PI3K/Akt and STAT3 pathways. This argues for upstream targeting of the PDGFRα given the redundancy of oncogenic pathways in PTC, especially in patients whose tumors over-express this tyrosine kinase receptor.

## INTRODUCTION

The global incidence of thyroid cancer has risen steadily over the last 40 years and in many countries it is expected to surpass colorectal cancer to become the fourth leading cancer diagnosis by 2030 [1, 2]. This increase is largely attributable to a tripling in the incidence of papillary thyroid cancer (PTC), which accounts for about 90% of thyroid cancers [1, 3]. PTCs have high propensity for nodal metastasis that usually involve surgical management and radioactive iodine ablation. For metastatic tumors, treatment is often more challenging due to inherent resistance to therapy, thus necessitating repeated surgical intervention or ablative treatments that impact patient quality of life with limited improvements in survival [3–6].

Randomized clinical trials have examined a repertoire of kinase receptor inhibitors for their ability to slow disease progression in patients failing surgical or radioactive iodine ablation treatment. In most cases, as reviewed by Gruber and Colevas [7], the responses to BRAF and MAPK blockade have been transient and there is substantial risk of toxicities and adverse events during therapy, limiting their widespread use. Clearly, targeted therapy selection demands a comprehensive understanding of the pathologic consequences of aberrant tyrosine kinase receptor activity in metastatic PTCs.

PDGFRs drive normal embryonal maturation by promoting mesenchymal cell development [8]. PDGFRα is required for gastrulation, the formation of neural crests, CNS, gonads, lung, intestine, skin and skeleton, while angiogenesis and early hematopoiesis are probably mediated through PDGFRβ [8–10]. PDGFRβ is a common finding in many tissue isolates; however, the PDGFRα subunit is typically not expressed or is expressed at low levels in most normal tissues. Aberrant activation of PDGFRα signaling has been observed in several human cancers including ovarian, melanoma, gastrointestinal stromal tumors, glioblastoma, prostate, breast, lung, renal cell and sarcoma. A role for PDGFRα in thyroid cancer was documented by Zhang *et al.*, through a clear association between its expression and metastatic papillary thyroid disease [11]. Biopsies from nodal deposits exhibited high levels of PDGFRα, while benign tissue and even primary tumors without metastases showed minimum PDGFRα expression [11]. Oncogenesis in PTC has traditionally been defined by aberrant MAPK/Erk signaling, but little is known about the impact of PDGFRα on this pathway and other downstream ones in the context of metastatic PTC.

The current study explores the aggressive phenotypic alterations fueled by PDGFRα in human PTC cells and the downstream signaling cascades through which they are effected. Particularly, the migratory behavior and three dimensional architecture of PTC cells were examined in constructs lacking or overexpressing PDGFRα. Furthermore, specific inhibitors of the downstream signaling pathways, PI3K/Akt, MAPK/Erk, STAT3 and Wnt/β-catenin, were utilized to define their contributions to the PDGFRα-induced phenotypic changes. As a therapeutic strategy, targeted inhibition of PDGFRα activity in PTC cells was also examined.

## RESULTS

### Cytokine activation and specificity for PDGFR alpha and beta subunits in PTC

**Figure 1:**
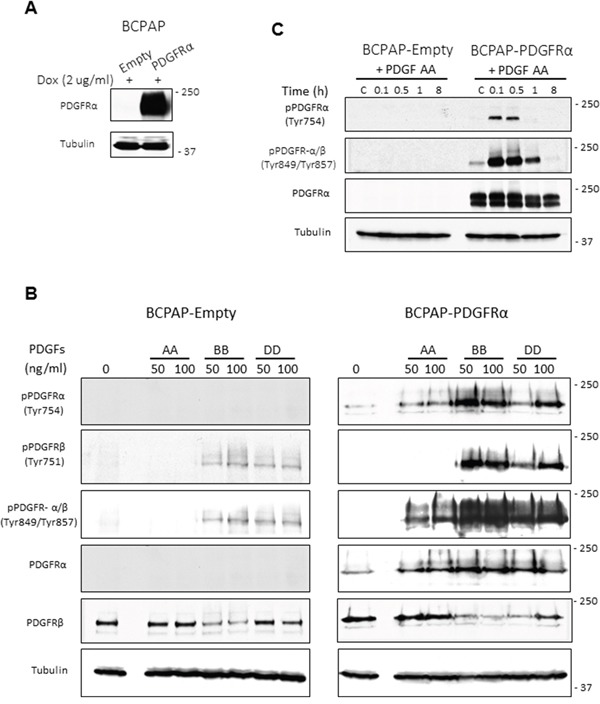
Inducible expression of PDGFRα in PTC cells and specificity of receptor activation Doxycycline-inducible expression of PDGFRα was achieved using a Tet-on system. Control cells containing only the empty vector (BCPAP-Empty) were also generated. **A.** PDGFRα protein expression was confirmed in cells after 48 h treatment with 2 μg/ml doxycycline (Dox). **B.** BCPAP-Empty cells (expressing PDGFRβ only) and BCPAP-PDGFRα cells (with both PDGFR –α and β), were serum-starved for 24 h, stimulated for 6 min with the PDGF ligands AA, BB and DD, then phosphorylation was detected for PDGFRα and PDGFRβ. **C.** Serum-starved BCPAP-Empty and BCPAP-PDGFRα cells were treated or untreated with 50 ng/ml PDGF-AA and cell lysates were collected at the indicated times. The time course for PDGFRα phosphorylation on Tyr754 and Tyr849 was analysed by western blotting. Tubulin was used as the loading control.

The PDGF ligands bind to and activate PDGFRα and PDGFRβ with different specificities, as reviewed by Heldin [9]. To confirm this in PTC cell lines, receptor activation in response to the ligand isoforms AA, BB and DD was assessed. PTC cell lines lacking or expressing PDGFRα on the same genetic background were compared to understand how PDGFRα drives changes in cell phenotype. Although BCPAP cells express PDGFRβ receptor as expected [11], they lack expression of PDGFRα (Supplementary Figure S1A). These cells were stably transduced with lentiviral vectors to express PDGFRα (BCPAP-PDGFRα) under doxycycline induction (Figure 1A). Control cells (BCPAP-Empty) contained only the empty vector. We also examined native 8305C cells that express PDGFRα.

PDGF-BB activates PDGFRα and PDGFRβ, whereas PDGF-AA preferentially activates PDGFRα with no effect on PDGFRβ (Figure 1B). Ligands BB and DD induced PDGFRβ phosphorylation whether it was expressed alone (BCPAP-Empty) or with PDGFRα (BCPAP-PDGFRα). PDGF-BB exhibited the same pattern of interaction with PDGFRα, whereas PDGF-DD did not activate PDGFRα when it was expressed alone as seen in 8305C (Supplementary Figures S1A and S1B). Thus PDGF-AA and -BB will activate PDGFRαα receptor homodimers, whereas DD and BB will activate ββ receptor homodimers as well as the αβ receptor heterodimers in PTC cells. In addition, 50 ng/ml of the ligands was sufficient to produce considerable levels of receptor phosphorylation (Figure 1B). Time dependent activation of PDGFRα in response to 50 ng/ml PDGF-AA is also shown in Figure 1C. As indicated by the phosphorylation of residues Tyr754 and Tyr849, PDGFRα activation peaks between 6 to 30 min of PDGF-AA treatment but disappears after 8 h of exposure.

### PDGFRα confers a branched 3D morphology and migratory phenotype

**Figure 2:**
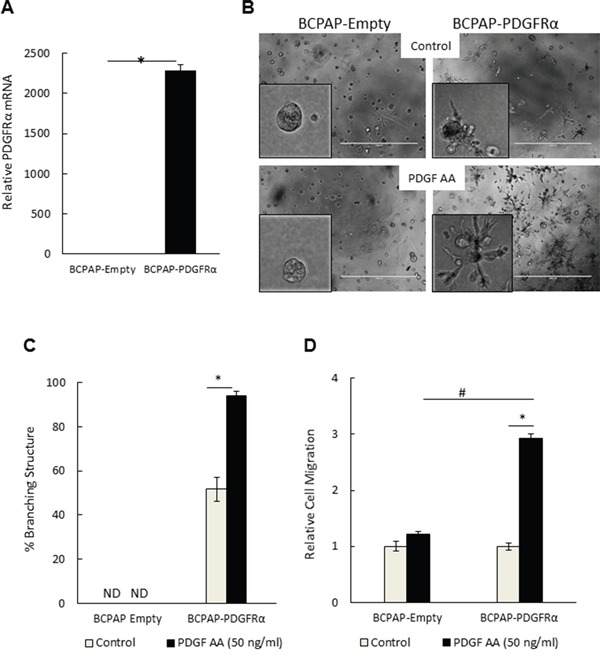
PDGFRα expression and activation conferred an invasive-like, branching 3D morphology and a migratory phenotype on papillary thyroid cancer cells **A.** Relative PDGFRα mRNA expression in BCPAP-Empty and BCPAP-PDGFRα cells after 7 days of 3D culture. **B-C.** BCPAP-Empty and BCPAP-PDGFRα cells were cultured in Matrigel for 7 days in the absence (control) or presence of 50 ng/ml PDGF-AA. (B) Representative phase contrast images (bar = 1000 μm) show dramatic induction of a branching morphology upon PDGFRα expression (upper right) and activation with PDGF-AA (lower right). The left panels show the contrasting 3D morphology of the control BCPAP-Empty cells. (C) The number of branching structures was presented as a percentage of the total number of structures observed. ND denotes branching structures not detected. **D.** PDGF-AA-induced migration of BCPAP-PDGFRα cells was about 3 times greater compared to BCPAP-Empty cells. For relative quantitation, the value of 1 was arbitrarily assigned to BCPAP-Empty cells. Results in C and D are means ± SEM, n = 6. Significant differences are indicated by *, p< 0.05 compared with untreated control cells, and #, p < 0.05 compared with corresponding samples of control BCPAP-Empty cells.

Given the specificity of PDGF-AA for the PDGFRα subunit, we further unraveled the phenotypic consequences of preferentially activating PDGFRα with this ligand. Using 3D Matrigel culture systems to mimic physiological conditions [12, 13], we investigated the effect of PDGFRα expression and activation on cell morphology by comparing BCPAP-Empty and BCPAP-PDGFRα cells. Figure 2A illustrates the over-expression of PDGFRα mRNA in BCPAP-PDGFRα cells relative to BCPAP-Empty cells after growing in 3D Matrigel matrix for 7 days. BCPAP cells expressing PDGFRα exhibited a highly branched 3D architecture with membrane protrusions (52% ± 5.5 of structures were branched) (Figures 2B and 2C). Treatment with 50 ng/ml PDGF-AA potentiated the formation of the branched structures by ~2-fold (94% ± 2). In contrast, control cells lacking PDGFRα formed dense and compact spheroids with no branching in the absence or presence of PDGF-AA. In agreement with this, 8305C cells, which natively express PDGFRα showed branched 3D architecture that was amplified by PDGF-AA supplementation, whereas branching was undetectable in KTC1 cells, which lack the receptor (Supplementary Figures S1A and S2). As another measure of metastatic potential, we assessed the ability of varying cell lines with and without PDGFRα to migrate by Boyden Chamber Assay. PDGF-AA stimulated the migration of BCPAP-PDGFRα cells by about 3-times more than the control BCPAP-Empty cells (Figure 2D). There was no significant difference in basal migration between both cell groups in the absence of PDGF-AA.

We further assessed the effect of specifically blocking PDGFRα activity using Crenolanib, a small molecule inhibitor of PDGFRα. The sensitivity of both BCPAP-Empty and BCPAP-PDGFRα to the Crenolanib concentrations used in this study is documented in Supplementary Figure S3. Both cell types responded similarly to Crenolanib treatment. While 1 μM Crenolanib, blocked cell growth by approximately 40%, no significant inhibition was observed with 0.1 μM and 0.5 μM.

**Figure 3:**
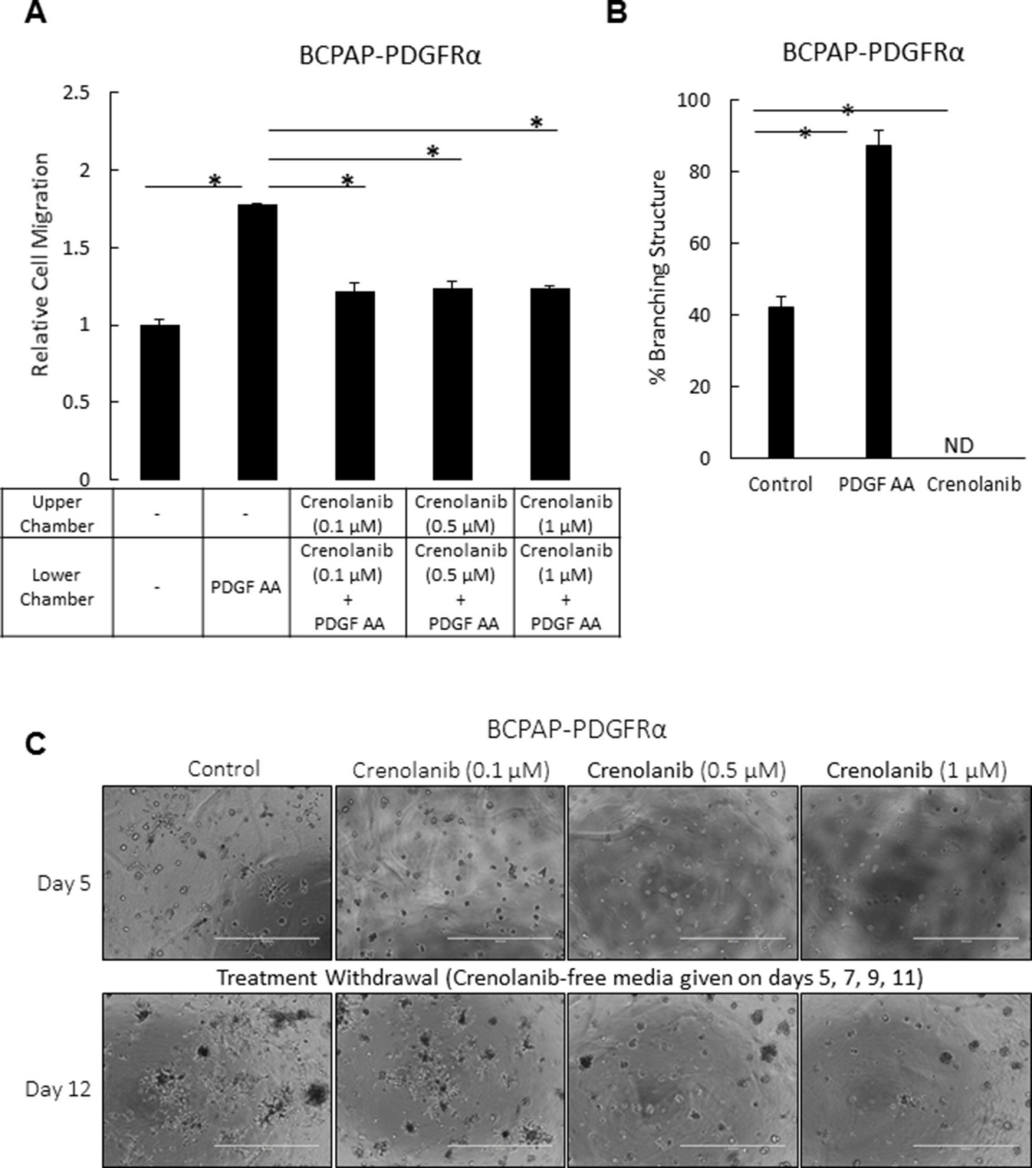
Inhibition of PDGFRα activation impedes cell migration in PTC cells and abrogates the transforming effect of PDGFRα on the 3D architecture **A.** The migration of serum-starved BCPAP-PDGFRα cells over 4 h in response to the gradient created by PDGF-AA (50 ng/ml) was quantified in the absence or presence of the indicated doses of Crenolanib. Untreated controls without Crenolanib and PDGF-AA were included. Results are means ± SEM, n = 8. Significant differences are indicated by *, p < 0.05. **B.** To assess the effect of blocking PDGFRα activity on the formation of the branched architecture, PDGFRα-expressing cells were grown in 3D culture for 7 days in the absence or presence of 1 μM Crenolanib or 50 ng/ml PDGF-AA. Results are means ± SEM, n = 6. *p < 0.05 compared to no treatment control. ND denotes branching structures not detected. **C.** The abrogative effect observed with Crenolanib was assessed for its reversibility over the indicted range of concentrations. PDGFRα-expressing cells were grown in 3D Matrigel culture in the absence (control) and presence of Crenolanib. Treatment was withdrawn after 5 days of propagation by feeding the cells with Crenolanib-free medium on days 5, 7, 9 and 11. Phase contrast images were acquired on days 5 and 12. Scale bars are 1000 μm. Higher magnification of these images are shown in Supplementary Figure S5.

Crenolanib blocked PDGF-AA-induced migration in serum-starved BCPAP-PDGFRα cells (Figure 3A). Disrupting the activation of PDGFRα also abrogated the formation of branched 3D structures in the PDGFRα-expressing cells. The 7-day timeline chosen for assessing the effect of Crenolanib on the branching morphology was the time taken for the BCPAP- PDGFRα cells to exhibit maximum branching, as observed in preliminary experiments without drug treatments. With 1 μM Crenolanib, the branched structures were undetectable compared to the untreated and PDGF-AA-treated cells in which the percentage of branched structures was 42% ± 3 and 87% ± 5, respectively (Figure 3B). In these PDGFRα-expressing cells, Crenolanib caused a complete architectural transformation to the compact, dense 3D spheres similar to those formed by cells lacking PDGFRα expression (Figure 2B). Next, we checked if the effect observed with Crenolanib was reversible. BCPAP cells expressing PDGFRα were exposed to different concentrations of Crenolanib for 5 days, after which the treatment was withdrawn. Following the initial 5-day treatment, BCPAP-PDGFRα cells exhibited a dense, spherical morphology with the branching undetectable at all Crenolanib concentrations used (Figure 3C). Untreated control cells exhibited the branched morphology as expected. A strong appearance of the branched structures was observed 7 days after the withdrawal of Crenolanib (0.5 μM and 1 μM). At higher concentrations, the compact, spherical morphology induced by Crenolanib treatment was maintained (Figure 3C).

### PDGFRα drives invadopodia formation through cytoskeletal rearrangement and upregulation of epithelial-mesenchymal transition markers Snail and Slug

Certainly, PDGFRα drives a remarkable change in cellular morphology, perhaps through the epithelial to mesenchymal transition (EMT) of cells, as a prerequisite to cancer cell dissociation, invasion, and metastasis. Therefore, consistent with cells undergoing EMT, we sought to establish if PDGFRα induced a reorganization of the cytoskeletal architecture, and possibly the biogenesis of invadopodia for directional migration [14–18]. We also assessed the levels of major protein effectors of EMT following the expression of PDGFRα.

**Figure 4:**
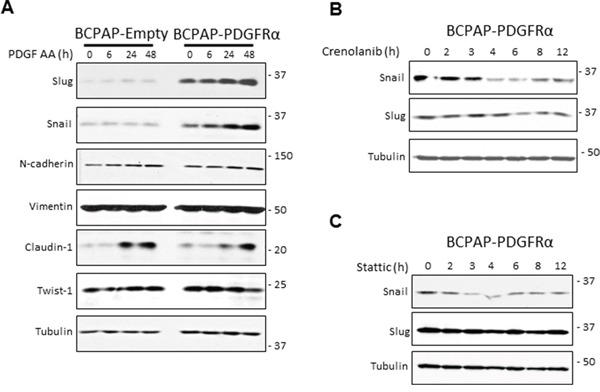
PDGFRα sensitizes cells to epithelial mesenchymal transition **A.** BCPAP cells lacking and expressing PDGFRα were grown as monolayers, serum-starved for 24 h, and stimulated with 50 ng/ml PDGF-AA for the indicated duration. Changes in the expression of EMT–associated makers, Slug, Snail, N-cadherin, Vimentin, and Twist-1 were monitored by the Western blotting with tubulin as the loading control. **B.** BCPAP- PDGFRα cells were serum-starved for 24 h and exposed to 1 μM Crenolanib for the times indicated. Cell lysates were probed for Snail and Slug expression. **C.** Similar experiments were performed using 2.5 μM Stattic to block STAT3 activation and the expression of Snail and Slug was monitored.

PDGFRα expression dramatically enhanced the protein levels of the transcription factors Snail and Slug, whose levels increased further in response to PDGF-AA stimulation (Figure 4A). Moreover, 1 μM Crenolanib caused a depletion of Snail protein expression in BCPAP-PDGFRα cells in a time-dependent manner, first noticeable after 4 h of treatment (Figure 4B). The repressive effect on Slug expression was modest and first evident after 6 h of Crenolanib treatment. Also, since STAT3 signaling seems to be important for the transformations in PDGFRα-expressing BCPAP cells, we examined the effect of blocking STAT3 activation. STAT3 inhibition with Stattic (2.5 μM) repressed Snail protein expression while there was no effect on Slug expression in BCPAP-PDGFRα cells (Figure 4C). There were no significant differences in the expressions of N-cadherin, Twist-1, and vimentin between cells lacking or expressing PDGFRα with or without PDGF-AA stimulation (Figure 4A).

**Figure 5:**
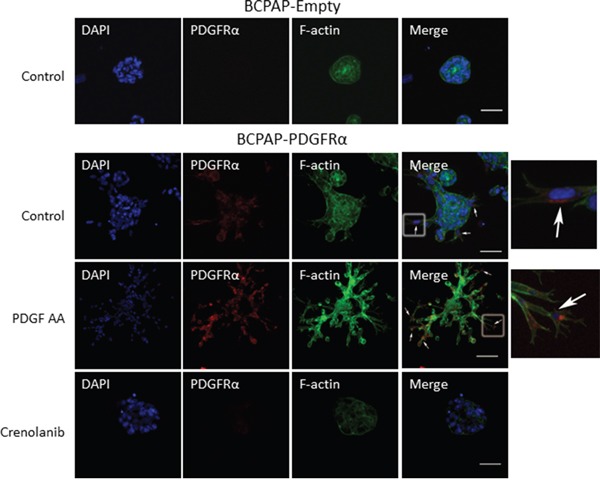
Crenolanib abrogates the PDGFRα-induced accumulation of F-actin associated with invadopodia formation BCPAP cells lacking or expressing PDGFRα were grown in 3D culture in the absence or presence of 1μM Crenolanib or 50 ng/ml PDGF-AA. Immuno-fluorescent staining of PDGFRα (red), F-actin (green) and DAPI (blue) was performed after 7 days in culture. Strong PDGFRα expression was seen mostly in cells at the tips of the protrusions (white arrows). Boxed regions in the merged images are enlarged and shown on the fifth panel. Scale bars are 50 μm.

Having established a link between PDGFRα activation and the expression of EMT associated markers, Slug and Snail, we determined if the protrusions observed with PDGFRα expression and activation exhibit components of invadopodia. BCPAP cells lacking or expressing PDGFRα were grown in 3D culture and the accumulation of F-actin, a major structural component of mature invadopodia [19] was examined by immunofluorescence. Relative to cells lacking PDGFRα, increased F-actin staining was observed with the formation of branched structures when PDGFRα was expressed and activated with PDGF-AA (Figure 5). The rich expression of F-actin in the protrusions is strongly characteristic of invadopodia. It is noteworthy that strong PDGFRα expression was seen mostly in cells at the tips of the protrusions (white arrows; Figure 5). Then, we checked if PDGFRα inhibition with Crenolanib affects F-actin expression. Relative to untreated and PDGF-AA-treated PDGFRα-expressing cells; those treated with Crenolanib displayed a large reduction in F-actin staining intensity as they lost the invadopodia-like projections (Figure 5). A significant loss in PDGFRα expression was also observed with Crenolanib treatment.

**Figure 6:**
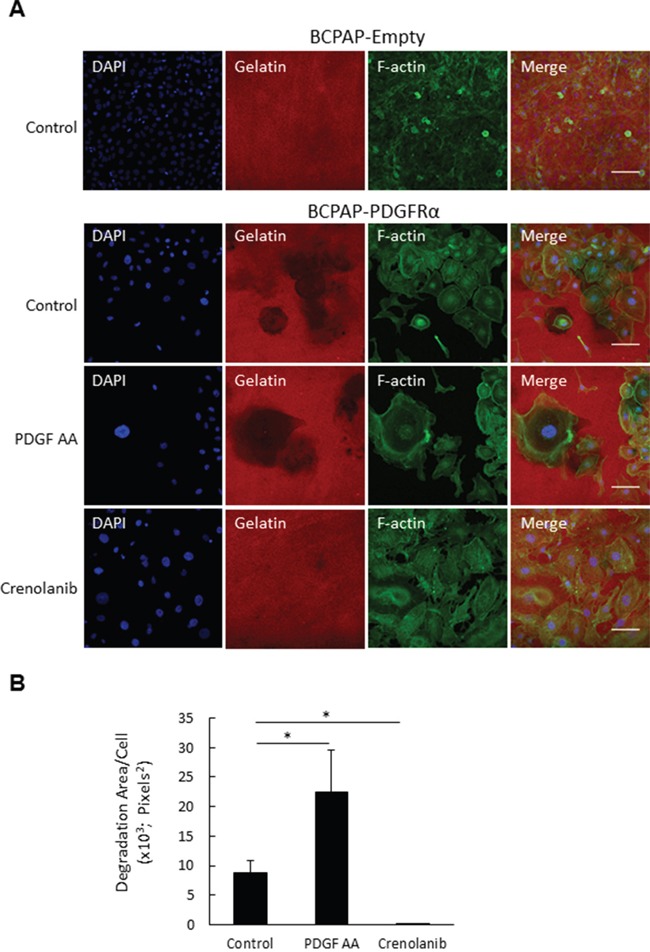
PDGFRα activation increases invadopodia degradation activity **A.** BCPAP-Empty and BCPAP-PDGFRα were plated on cy3-conjugated gelatin and cultured with or without PDGF-AA and Crenolanib for 4 days. Cells were stained for F-actin (green) and nuclei (blue) and imaged at 20X objective magnification at 5 fields of view per well. Representative image sets are shown for all treatments (scalebar = 100 μm). Dark patches on cy3-conjugated gelatin (red) indicate areas of degradation. **B.** Quantification of mean degradation area per cell as described in the Materials and Methods. *, p < 0.05.

To establish whether the PDGFRα-induced protrusions are functional invadopodia, BCPAP-Empty and BCPAP-PDGFRα cells were assessed for their gelatinase ability. As shown by the immunofluorescent images, gelatin degradation was seen in BCPAP-PDGFRα cells while BCPAP-Empty cells showed no gelatinase ability (Figure 6A). The degradation area increased by 3-fold when BCPAP-PDGFRα cells were treated with PDGF-AA compared to the untreated (control) cells (Figure 6B). Crenolanib, almost completely abolished the degradative ability of BCPAP-PDGFRα cells, and the degradation area significantly reduced by ~8-fold relative to the control cells (Figure 6B). As delineated by F-actin staining, BCPAP-PDGFRα cells with high gelatinase activity seemed to be larger than the BCPAP-Empty cells with no degradative activity (Figure 6A).

### PDGFRα signaling and its downstream effector pathways in PTC cells

After observing the dramatic phenotypic alterations associated with PDGFRα, we investigated the downstream pathways that effect its pro-metastatic cues. The MAPK/Erk, PI3K/Akt, STAT3, and the Wnt/βcatenin pathways were selected for assessment due to their well-documented link to thyroid cancer development and progression [20–23]. Pathway activation was assessed in BCPAP-PDGFRα cells after the homodimeric form of PDGFRα was stimulated with PDGF-AA (given the results in Figure 1B) and specifically blocked with Crenolanib [24, 25]. After pretreatment with various concentrations of Crenolanib (0.1 μM, 0.5 μM, and 1 μM) for 1 h, cells were exposed to PDGF-AA for 6 min. The phosphorylation levels of Erk, Akt, STAT3 and GSK3β served as indicators for Erk/MAPK, PI3K/Akt, STAT3, and the Wnt/βcatenin pathway activation, respectively. Upon Wnt pathway activation, GSK3β is inactivated by its phosphorylation on Ser9, allowing for cytoplasmic accumulation of β-catenin, which is the primary effector of the Wnt/βcatenin pathway. β-catenin promotes the transcription of Wnt-target genes [23].

**Figure 7:**
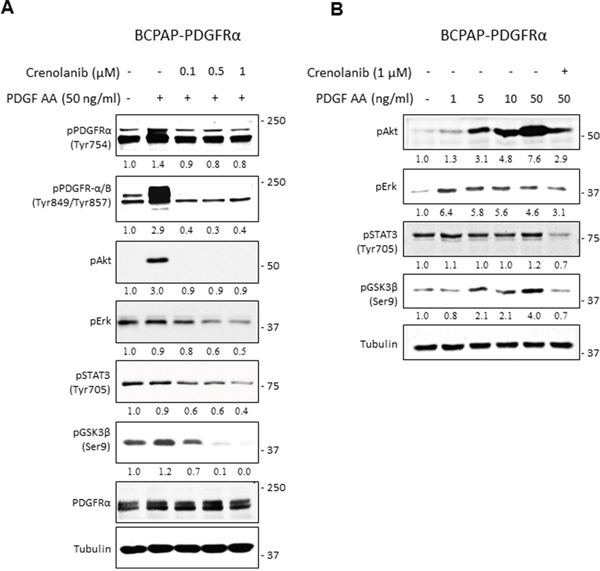
PDGFRα signaling and its downstream effector pathways in PTC cells **A.** Pharmacological inhibition of PDGFRα activity with Crenolanib was confirmed by western blot. BCPAP-PDGFRα cells were serum-starved for 24 h, pretreated with or without the indicated Crenolanib doses for 1 h, followed by 6 min stimulation with 50 ng/ml PDGF-AA. Cells unexposed to both Crenolanib and PDGF-AA served as control. Cell lysates were subjected to western blot analyses and equal protein loading was confirmed with tubulin expression. **B.** Serum-starved BCPAP-PDGFRα cells were pretreated with or without 1 μM Crenolanib for 1 h and stimulated with or without the indicated concentrations of PDGF-AA for 15 min followed by western blot analyses of Erk, Akt, STAT3 and GSK3β phosphorylation. Tubulin was used as the loading control.

Increased PDGFRα phosphorylation on residues Tyr754 and Tyr849, which corresponds with significant upregulation of Akt phosphorylation was observed with PDGF-AA treatment (Figure 7A). When cells were pretreated with Crenolanib, PDGFRα phosphorylation was suppressed to comparable levels while Akt phosphorylation was completely blocked with all Crenolanib concentrations (Figure 7A). The induction of Erk, STAT3 (Tyr705) and GSK3 (Ser9) phosphorylation after this short exposure to PDGF-AA was very weak. However, Crenolanib significantly inhibited their phosphorylations in a dose-dependent manner. When BCPAP-PDGFRα cells were exposed to increasing concentrations of PDGF-AA for 15 min, phosphorylated Akt and GSK3β increased with increasing doses of PDGF-AA (Figure 7B). Whereas, PDGF-AA stimulated increase in phosphorylated Erk and STAT3 was weak and independent of dose.

### STAT3 signaling plays an important role in the PDGFRα-associated phenotypes

**Figure 8:**
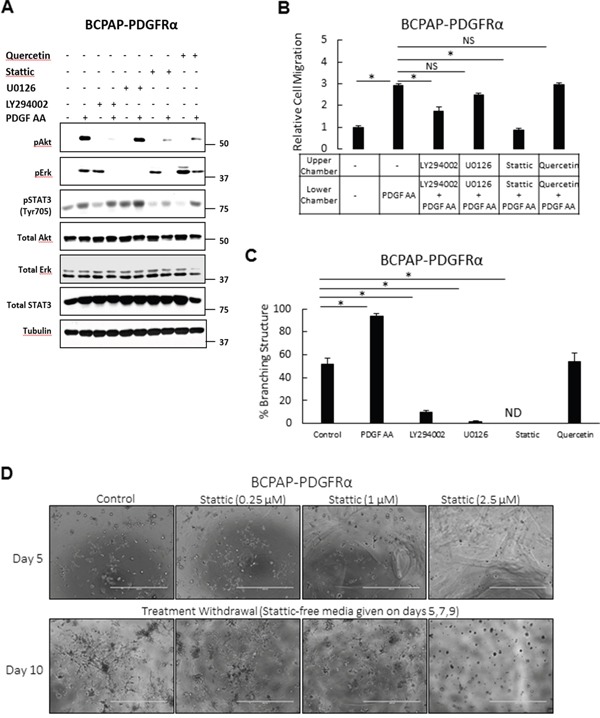
Effects of inhibiting signaling pathways on the PDGFRα-associated phenotypes To assess the relative roles of the Erk/MAPK, PI3K/Akt, STAT3, and Wnt/βcatenin pathways in establishing the PDGFRα-associated phenotypes, these pathways were pharmacologically blocked with U0126 (10 μM), Ly294002 (10 μM), Stattic (2.5 μM) and Quercetin (10 μM), respectively. **A.** Cells expressing PDGFRα were serum-starved for 24 h, pretreated with the inhibitors for 1 h, and then stimulated with 50 ng/ml PDGF-AA for 6 min. Pathway blockade was confirmed by monitoring the phosphorylation levels of Erk, Akt, and STAT3 on western blots **B.** PDGFRα-induced migration was quantified in BCPAP-PDGFRα cells following treatment with the inhibitors. **C.** The proportion of branching structures formed in the presence of the inhibitors was assessed in BCPAP-PDGFRα cells after propagation in 3D culture for 7 days. ND denotes branching structures not detected. **D.** The inhibitory effect of Statttic on the PDGFRα-induced 3D morphology was assessed for its reversibility over the indicated concentration range. Treatment of PDGFRα expressing cells with Stattic was discontinued after 5 days, and cells were allowed to grow for another 5 days. Images were acquired on days 5 and 10 (scale bars; 1000μm). Higher magnification of these phase contrast images are shown in Supplementary Figure S6. Quantitative results (B and C) are expressed as means ± SEM, n = 6. Significant differences are indicated by * when p < 0.05, NS =Not significant.

The role of the Erk/MAPK, PI3K/Akt, STAT3, and Wnt/βcatenin pathways in establishing the PDGFRα-associated phenotypes were studied by selective inhibition with U0126, LY294002, Stattic and Quercetin, respectively. In cells expressing PDGFRα, PDGF-AA-induced phosphorylation of Akt, Erk, and STAT3 was abrogated by LY294002, U0126, and Stattic, respectively (Figure 8A). Corresponding quantification of these phospho-protein levels in response to the inhibitors is shown below the bands. Stattic had a considerable inhibitory effect on PDGF-AA-mediated Akt phosphorylation. Quercetin, a potent inhibitor of β-catenin transcriptional activity [26] significantly reduced ligand-activated Akt phosphorylation levels. Surprisingly, treatments with LY294002, Stattic, and Quercetin strongly induced Erk phosphorylation in a PDGF-independent manner.

While the inhibition of PI3K/Akt, and STAT3 pathways reduced PDGF-AA-stimulated migration of PDGFRα expressing cells, blockade of MAPK/Erk and Wnt/βcatenin had no significant effect (Figure 8B). STAT3 blockade produced the most significant anti-migratory effect such that the migratory ability of Stattic-treated cells was comparable with that of untreated control cells.

Blockade of the MAPK/Erk, PI3K/Akt and STAT3 pathways in PDGFRα expressing cells significantly reverted the formation of branched 3D structures in culture, while the effect of Wnt/βcatenin pathway inhibition was inconsequential (Figure 8C). The inhibitor concentrations used had minimal effect on cell viability (Supplementary Figure S4). Given that STAT3 blockade produced a complete morphology reversion with no detectable branched structure, we further checked if this strong inhibitory effect with Stattic was reversible over a concentration range of 0.25 μM, 1 μM and 2.5 μM. Treatment of PDGFRα expressing cells with Stattic was discontinued after 5 days, and cells were allowed to grow for another 5 days. Branching was blocked in a dose-dependent manner; with the highest inhibitory effect seen with 2.5 μM Stattic (Figure 8D). After withdrawal, branched structures returned at all concentrations used, however the strongest appearance was observed in cells treated with 0.25 μM Stattic.

### PDGFRα stimulates nuclear accumulation of STAT3

Given that STAT3 signaling was crucial for establishing the PDGFRα-associated phenotypes, the relationship between PDGFRα stimulation and STAT3 activity was further established. STAT3 phosphorylation (Tyr705), homodimerization and nuclear translocation are required to regulate the expression of its target genes [27]. Since PDGF-AA-induced activation of PDGFRα led to enhanced STAT3 (Tyr705) phosphorylation (Figure 8A), subsequent nuclear translocation and accumulation of phosphorylated STAT3 (Tyr705) was expected.

**Figure 9:**
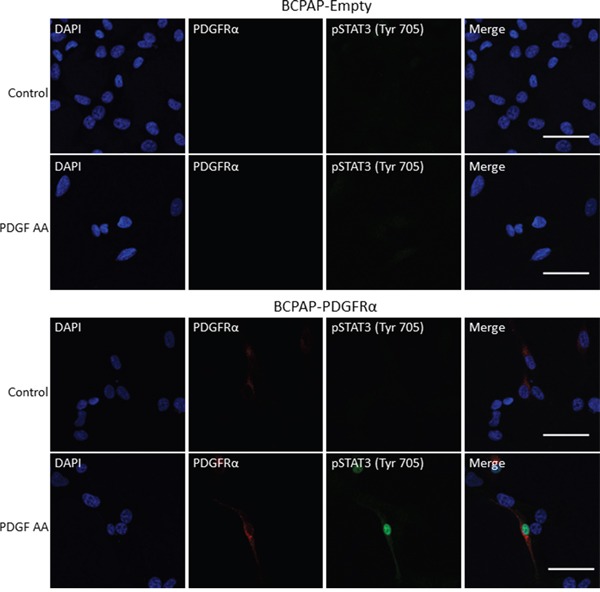
PDGFRα activation induces nuclear accumulation of pSTAT3 BCPAP-Empty and BCPAP-PDGFRα cells were seeded onto 8-well chamber slides overnight, serum starved for 24 h and treated with or without PDGF-AA (50 ng/ml) for 20 min. The cells were prepared for immunofluorescence staining of PDGFRα (red), pSTAT3 (green), and the nucleus (blue) as described in Materials and Methods. Scale bars are 50 μm.

To determine if this is the case, starved BCPAP-Empty and BCPAP-PDGFRα cells were treated with PDGF-AA for 20 min, fixed and subjected to immunofluorescence staining for pSTAT3 (Tyr705), PDGFRα and the nucleus. pSTAT3 expression was not detectable in the nucleus and cytoplasm of untreated BCPAP-Empty and BCPAP-PDGFRα cells, but was observed in the nucleus of PDGFRα-expressing cells within 20 min of PDGF-AA stimulation (Figure 9). Nuclear and cytoplasmic staining for pSTAT3 (Tyr705) was also absent in BCPAP-Empty cells treated with PDGF-AA.

## DISCUSSION

The management of metastatic PTCs is challenging because of significant morbidity as a result of repeated surgical resections or high-dose radioactive iodine treatments, which become the only recourse upon recurrence [5]. We demonstrate that specific blockade of PDGFRα is sufficient to reverse the aggressive phenotypic changes seen in PTC cells and this may represent an advance in therapy for those patients with progressive, metastatic disease.

In this report, we present compelling mechanistic evidence that PDGFRα drives an aggressive cellular phenotype as a consequence of epithelial to mesenchymal transition through substantial elevation of Snail and Slug. These phenotypic changes observed *in vitro* parallel those findings documented in clinical studies of PTC specimens whereby PDGFRα is strongly associated with metastatic disease [11]. The gene expression changes that provoke the switch from the epithelial to mesenchymal phenotype, are mediated by regulators like Snail and Slug, whose expression is activated early in EMT [28]. During the EMT, non-motile, polarized epithelial cells shed their intracellular adhesion molecules to become individual, non-polarized, motile mesenchymal cells. The EMT is a well-regulated physiological process in embryogenesis where it promotes the mesenchymal cell development. However, the central dogma is that aggressive cancer cells usurp the EMT machinery, in other to fuel their metastatic spread [28]. The up-regulation of Snail and Slug with corresponding increases in aggressiveness has been observed in many human malignancies [29–31], including PTCs where they were detected in lymph node metastases [32]. However, it is noteworthy that the molecular algorithm associated with the EMT has an inherent flexibility and variation, which depends on the cell type, tissue context and signals that activate the transition [33]. This is a plausible explanation for the unaltered levels of the other EMT markers (N-cadherin, Twist-1 and vimentin), observed in the PDGFRα-expressing cells.

In addition to the changes in molecular signature, transition to the mesenchymal phenotype is usually accompanied by dramatic architectural alterations, which include cytoskeletal reorganization and the formation of invadopodia projections capable of extracellular matrix degradation [28]. We provide here the first evidence of a link between PDGFRα signaling and functional invadopodia in PTC cells. It is now widely accepted that invadopodia are vital for the major steps in the metastatic dissemination of cancer cells: local invasion of stromal tissues at the primary site, intravasation into the vasculature, and extravasation at distant sites [34]. PDGFRα-expressing cells displayed increased motility in response to a PDGF-AA gradient, indicating a migratory role for the invadopodia-like projections seen in 3D culture. This suggestion is founded on a previous observation that invadopodia are also involved in signal sensing and directional protrusion during chemotaxis [35]. Similar to our findings, Gotzmann et al., [36] reported that the PDGF-AA/PDGFRα signaling axis promotes tumor progression by enhancing the motility of hepatocytes undergoing EMT.

We further characterise the PDGFRα-induced protrusions as functional invadopodia, as judged by their gelatinase activity. The assessment of invadopodia-mediated ECM degradation by microscopy using fluorescently-labeled matrix proteins has emerged as the most prevalent technique. [37]. Overexpression of PDGFRα and its activation in PTC cells drastically increased the ability of the cells to degrade cy3-labeled gelatin matrix, while PDGFRα-deficient cells displayed null gelatinase activity. Tumor cells leverage the aptitude of the invadopodia protrusions for ECM degradation to overcome the physical barriers presented by the basement membrane, the interstitial matrix, and the endothelial cells during metastasis [38]. *In vitro* observations of enzymatic ECM degradation by invadopodia-forming cells have been made in cultures of melanoma, breast, prostate, as well as head and neck squamous cancer cells [38–41]. Similar to our findings, a positive association between EMT transcriptional regulators, Twist1 and Snail, as well as PDGFRα expression was shown to be necessary for invadopodia development in breast cancer cells [15]. *In vivo* evidence from animal models showing that invadopodia are crucial for successful metastasis has also been documented [14, 42]. Thus, our results suggest that PDGFRα promotes the biogenesis of functional invadopodia in PTC cells, as part of its metastasis-driving mechanism.

Given that PDGFRα induces a degradative, migratory phenotype in PTC cells, we investigated the downstream effectors of its pro-metastatic cues. PI3K/Akt, MAPK/Erk, and STAT3 were activated by PDGFRα in PTC cells. Blocking these pathways, especially STAT3, with pharmacological agents disrupted the invadopodia-like formations in PDGFRα-expressing cells, and reduced the number of migrating cells in 2D culture. These results strongly implicate the STAT3 pathway as the major effector of the pro-metastatic signals from PDGFRα, while the PI3K/Akt and MAPK/Erk pathways played lesser or adjunct roles. Our finding mirrors previous reports of constitutive STAT3 activation in response to aberrant upstream tyrosine kinase activities in a broad spectrum of human cancers [43]. More specifically, histological analysis showed increased expression and activation of STAT3 in the nodal deposits of metastasis PTCs [22]. These results directly challenge the canonical description of PTC as a MAPK/Erk-driven malignancy, and clearly demonstrate a vital role for the STAT3 and AKT pathways in driving this aggressive disease phenotype. The compensatory response of the MAPK/Erk pathway to the inhibition of the PI3K/Akt, STAT3 and Wnt/β-catenin pathways underscores the complex and cooperative crosstalk that exist amongst these pathways under pathologic conditions.

Targeted therapy of PTC has become the focus of many clinical studies with the development of potent inhibitors including imatinib, sunitinib, sorafenib, lenvatinib and nilotinib [24, 25, 44–48]. However, these tyrosine kinase inhibitors (TKI) lack specificity for PDGFRα and they act on a spectrum of tyrosine kinases. For instance, in addition to inhibiting PDGFRα, sunitinib inhibits PDGFRβ and the vascular endothelial growth factor receptors [10]. Crenolanib, a novel TKI, which targets and inhibits PDGFRα activity with high specificity, has been tested for its therapeutic efficacy in other malignancies [24, 25, 44]. Here, we propose that metastatic PTC may be treated with the focused pharmacological inhibition of PDGFRα using Crenolanib. Consistent with this hypothesis, Crenolanib, suppressed the levels of Snail and Slug and abrogated the aggressive phenotypes associated with PDGFRα. This is the first evidence suggesting that inhibiting the activation of PDGFRα could provide a potentially beneficial treatment for metastatic PTC patients whose tumors over-express this receptor. Moreover, we provide evidence for the activation of multiple pathways in metastatic PTC, indicating that upstream inhibition of PDGFRα activity presents a more rational therapeutic approach than the MAPK/Erk pathway blockade.

## MATERIALS AND METHODS

### Materials

PDGF ligands AA, BB and DD were purchased from Life Technologies (Grand Island, NY). Crenolanib was obtained from Selleckchem (Houston, TX). MAPK/Erk inhibitor U0126 and PI3K/AKT inhibitor LY294002 were purchased from Cell Signaling Technology (Beverly, MA). STAT3 inhibitor, Stattic, and Quercetin, an inhibitor of β-catenin transcriptional activity were obtained from Sigma-Aldrich (Oakville, ON, Canada). Inhibitor stock solutions were prepared by reconstitution in dimethyl sulfoxide (DMSO; Sigma-Aldrich) following the suppliers' instructions. Dilutions were performed in growth media to make the working concentrations.

### Cell culture

Human PTC cell lines BCPAP, 8305C and KTC1 were purchased from American Type Culture Collection (Rockville, MD) and cultured in high glucose Dulbecco's Modified Eagle Medium (DMEM; Life Technologies) supplemented with 10% fetal bovine serum (FBS; Sigma-Aldrich) under an atmosphere of 5% CO_2_ at 37°C.

### Establishment of stable cell lines

A doxycycline-inducible retrovirus system was used (Lenti-X Lentiviral Expression Systems; Clontech Laboratories, Inc., Mountain View, CA) to stably express human PDGFRα in BCPAP cells. These cells were first transduced with the LVX-Tet-On advanced lentivirus (Neo+) followed by selection in G418 (1 mg/ml). Resistant cells were then transduced with the LVX-Tight-Puro (Puro+) vector or sequence-verified derivatives expressing wild-type human PDGFRα cDNA, followed by selection in puromycin (2.5 μg/ml). cDNA expression was induced by addition of doxycycline (2 μg/ml) and protein expression was verified by immunoblotting for the total and phosphorylated forms of PDGFRα.

### Western blot and antibodies

Cells were washed with phosphate-buffered saline (PBS), and cell proteins were solubilized using RIPA buffer supplemented with 0.1 mM phenylmethylsulfonyl-fluoride, protease inhibitor cocktail (1:100; Sigma-Aldrich) and phosphatase inhibitor cocktail 2 (1:50; Sigma-Aldrich). Lysates were centrifuged at 20,000 g for 15 min to remove cell debris. Supernatants were collected and protein concentrations were determined using BCA Protein assay kit (Pierce, Thermo Fisher Scientific Inc., Rockford, IL). Protein samples were boiled for 10 min in SDS sample buffer, resolved by SDS-PAGE and transferred onto nitrocellulose membranes (Bio-Rad, Richmond, CA). Membranes were blocked for 1 h with 5% skimmed milk in Tris-buffered saline containing 0.1% Tween-20 (TBS-T) and subsequently incubated overnight at 4°C with primary antibodies. Membranes were washed thrice with TBS-T and incubated for 1 h at room temperature in the appropriate secondary antibody conjugated with horseradish peroxidise (Bio-Rad). Following three washes with TBS-T, protein bands were detected using a chemiluminescent detection kit (Pierce). Primary antibodies listed in Supplementary Table S1 were used at the indicated dilutions. Equal loading of protein was confirmed by γ-tubulin expression.

### Three-dimensional (3D) cell culture

Growth factor-reduced Matrigel (BD, Cambridge, MA), 130 μl was spread as a thick layer on 8-well chamber slides, and polymerized at 37°C for 15 min. Then cells grown as monolayer were trypsinized, re-suspended in the appropriate growth media containing with 2% Matrigel and plated on top of the pre-coated Matrigel. Cells (6000) were seeded into each well and growth medium was replenished every two days until colonies were established. In some experiments, colony development proceeded in the sustained presence of PDGF-AA (50 ng/ml), LY294002 (10 μM), U0126 (10 μM), Stattic (2.5 μM), Quercetin (10 μM) or Crenolanib (1 μM). Cells were fixed at indicated times with 4% paraformaldehyde and phase-contrast images were acquired using an AMG EVOS digital inverted microscope. The degree of branching was assessed using these images and quantified with Adobe Photoshop. A branching structure was defined as a colony having at least one process (branch) extending from its body. A fixed area was randomly selected by using the select tool, the number of colonies with branches were counted, and expressed as a percentage of the total number of colonies within the specified area. Values are means ± SE.

### Quantitative real-time PCR (qPCR)

First, using qPCR, the gene expression levels of PDGFRα in the BCPAP-Empty and BCPAP-PDGFRα cells grown in 3D culture was confirmed. The 3D structures formed were recovered from the Matrigel Matrix using the Corning Cell Recovery Solution (Bedford, MA) according to manufacturer's instructions. Briefly, the medium was removed, the Matrigel-embedded structures were washed 3 times with cold PBS and the Recovery Solution was added at volumes recommended by the manufacturer. The structures/gel were scraped into tubes and left on ice for 1 h and spun down at 300 g for 5 min at 4°C. Then, the released 3D structures were washed in ice-cold PBS twice, and processed for RNA extraction. Total RNA was isolated using the RNeasy Mini Kit (Qiagen, Hilden, Germany) and cDNA was synthesized with the High Capacity cDNA Reverse Transcription Kit (Applied Biosystems, Foster City, CA) according to the manufacturers' instructions. Two μg of RNA was reverse transcribed in a 20 μl reaction volume. qPCR amplification was performed with the resulting cDNA using the 7900HT fast real-time PCR system (Applied Biosystems). All primers were obtained from Integrated DNA Technologies (Coralville, IA): PDGFRα: 5′ TAGTGCTTGGTCGGGTCTTG-3′ (forward), 5′ TTCATGACAGGTTGGGACCG-3′ (reverse), GAPDH: 5′-GTCTCCTCTGACTTCAACAGCG-3′ (forward), 5′-ACCACCCTGTTGCTGTAGCCAA-3′ (reverse). The Ct values were estimated by the SDS2.3 software package (Applied Biosystems) and exported to RQ-manager v1.2 (Applied Biosystems) for relative quantitation. Three independent measurements of PDGFRα gene expression normalized to GAPDH were made. Relative expression was calculated with BCPAP-Empty cells as the calibrator and assigning it a PDGFRα gene expression value of 1.

### F-actin staining and fluorescent visualization

For F-actin staining, cells in 3D culture were fixed in 4% paraformaldehyde/PBS for 1 h, permeabilized with 0.5% Triton X-100/PBS for 10 min, washed in glycine/PBS for 10 min and blocked with 5% goat serum. To confirm PDGFRα-induced accumulation of nuclear pSTAT3, cells were seeded at 4,000 cells per well on 8-well chamber slides. The cells were serum-starved for 24 h and treated with or without PDGF-AA (50 ng/ml) for 20 min, fixed in 4% paraformaldehyde and permeabilized using ice-cold methanol. Samples were then incubated overnight at 4°C with the appropriate primary antibodies (PDGFRα, 1:800 and pSTAT3, 1:50) and Alexa 488-conjugated phalloidin for F-actin staining (Life Technologies). For visualization of the PDGFRα and pSTAT3, cells were further incubated with Alexa 594- and Alexa 488- conjugated secondary antibodies (Life Technologies) respectively for 1 h. Cell nuclei was counterstained with 4', 6-diamidino-2-phenylindole (DAPI) and samples were mounted with Prolong Gold Anti-fade reagent (Life Technologies). Images were taken with a Zeiss LSM 710 Axio Observer inverted 34-channel confocal microscope and analyzed with Zeiss Zen software.

### Invadopodia assay and fluorescent visualization

For the invadopodia assay, the QCM™ Gelatin kit (EMD, Millipore) was purchased and used according to the manufacturer's guidance. Briefly, to facilitate attachment of the cy3-conjugated gelatin, 8-well glass chamber slides were first coated with poly-L-lysine for 20 min, rinsed three times with PBS, incubated for 15 min in glutaraldehyde and washed three times with PBS. Gelatin was mixed at a 1:5 ratio (fluorescently-labeled unlabeled gelatin), 200 μL of the mixture was incubated in each well for 10 min, followed by three rinses in PBS. Disinfection with 70% ethanol for 30 min, wells were rinsed with PBS, and free aldehydes were quenched by the addition growth medium for 30 min. Then, BCPAP-Empty and BCPAP-PDGFRα cells were seeded at 10, 000 cells/well and cultured for 4 days in the presence of PDGF-AA (50 ng/ml) or Crenolanib (1 μM). Untreated cells without both treatments were also cultivated.

For immunofluorescent imaging of invadopodia activity (gelatin degradation), cells were fixed for 30 min at RT in 4% paraformaldehyde, rinsed twice with fluorescent staining buffer (PBS with 2% blocking serum and 0.25% Triton X-100 for cell permeabilization). To assess F-actin expression, cells were blocked in the fluorescent staining buffer for 1h, incubated with Alexa-488 conjugated phalloidin for 45 min, the cell nuclei were counterstained with 4', 6-diamidino-2-phenylindole (DAPI) for 5 min and samples were mounted with Prolong Gold Anti-fade reagent (Life Technologies). Images of gelatin degradation and F-actin staining were taken with a Zeiss LSM 710 Axio Observer inverted 34-channel confocal microscope 20X objective lens and analyzed with Zeiss Zen software. Invadopodial activity was identified as areas of gelatin degradation characterized by loss of red fluorescence. The Metamorph software (Molecular Devices) was used to quantify gelatin degradation. Gelatin degradation was measured as the average area of non-fluorescent pixels per field. 5 random fields were imaged per condition and each independent experiment was performed at least 3 times and averaged.

### Trypan blue exclusion and MTS assays

To assess the effect of LY294002, U0126, Stattic, Quercetin, or Crenolanib on proliferation, cells were seeded at 35,000 cells per well in 12-well culture plates. After 24 h of treatment, cells were trypsinized, stained with trypan blue (Sigma) and counted with a hemocytometer. Results are expressed as total numbers of viable cells. The MTS assay (Promega, Madison, WI, USA) was also performed to assess the cytotoxicity of Crenolanib at various concentrations after 5 days of treatment. Results are expressed as a percentage of untreated controls.

### Migration assay

Cell migration was assessed using 96-well Boyden Chamber apparatus [49]. Cells were serum-starved overnight and detached with PBS containing 2 mM EDTA and 0.1% BSA, pH 7.4. Cells were washed three times with serum-free DMEM and suspended in DMEM containing 0.1% BSA to achieve a concentration of 300,000 cells/ml. The top chambers were loaded with 100 μl of cell suspension, and the bottom chambers were filled with DMEM containing the agonist (50 ng/ml PDGF-AA). The top and bottom chambers were separated with polycarbonate membrane containing 8 μm pores, which was coated with 10 μg/ml fibronectin overnight at 4°C. Cell migration was measured over 4 h at 37°C. Cells attached to the membrane were fixed with methanol. Then, cells on the top surface of the membrane were wiped off while those that migrated across the membrane to the bottom were stained with crystal violet and quantified with an Odyssey infrared imaging system (LI-COR Biosciences).

### Statistical analyses

Results are expressed as means ± SEM. Two-tailed student's t-test, and one-way ANOVA with a Bonferonni post-hoc test were used to test significance as appropriate. *p*-values <0.05 were considered to be significant.

## SUPPLEMENTARY FIGURES AND TABLE


