# Supplementary material for: Platelet derived growth factor receptor alpha mediates nodal metastases in papillary thyroid cancer by driving the epithelial-mesenchymal transition
Source: Oncotarget. 2016 Nov 11;7(50):83684–700. doi: 10.18632/oncotarget.13299 (PMC5347797; doi:10.18632/oncotarget.13299)
Supplement: Supplementary file 1 [file oncotarget-07-83684-s001.pdf]

Platelet derived growth factor receptor alpha mediates nodal metastases in papillary thyroid cancer by driving the epithelial-mesenchymal transition

SUPPLEMENTARY FIGURES AND TABLE

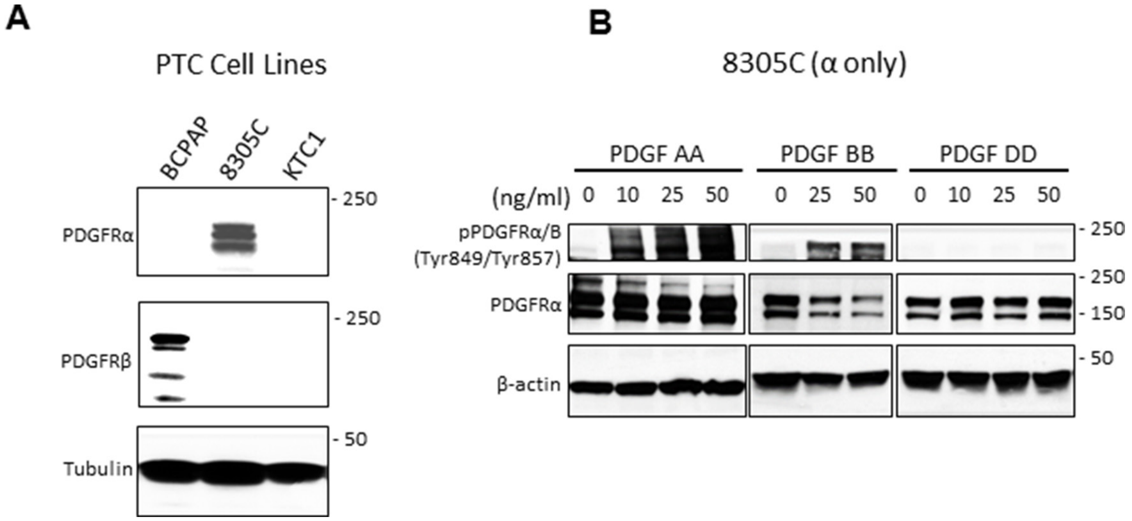

Supplementary Figure S1: A. Western blot analysis showing the native expression profile of PDGFR -α and β in PTC cell lines BCPAP, 8305C and KTC1. **B.** 8305C cells expressing PDGFRα only, was stimulated for 6 min with the PDGF ligands AA, BB and DD and probed for PDGFRα phosphorylation.

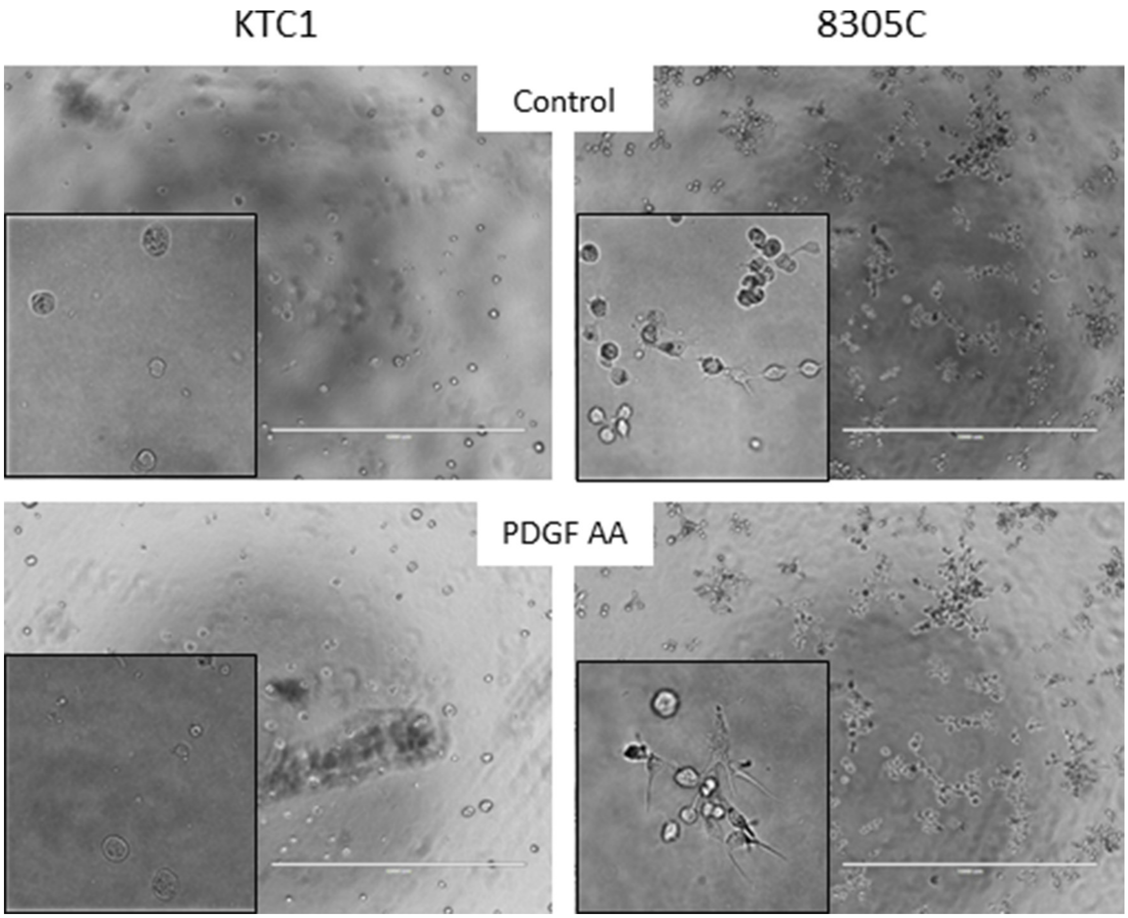

Supplementary Figure S2: PDGFRα promotes branching in 3D culture. 8305C cells (with native PDGFRα expression) and KTC1 cells (without PDGFRα expression) were grown in 3D Matrigel culture for 7 days. While 8305C cells exhibited branched 3D morphology, KTC1 cells produced dense spheroids in the absence and presence of PDGF-AA. Scale bars are 1000 μm.

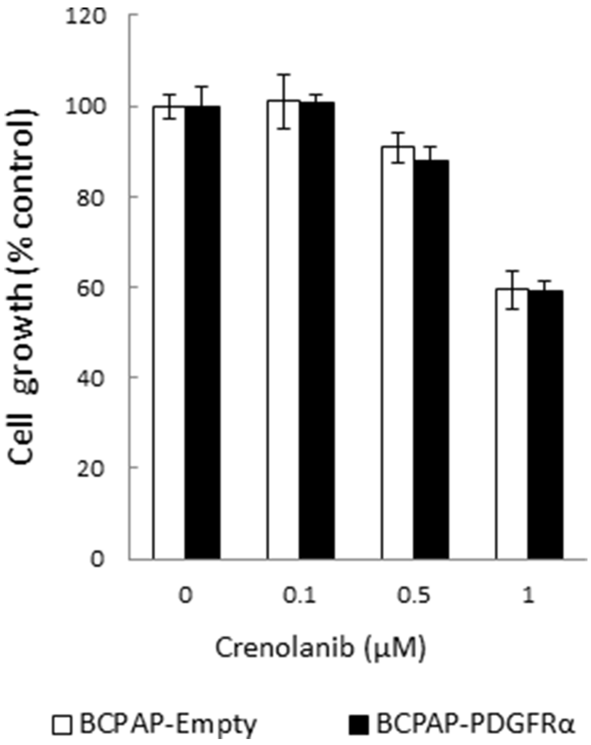

Supplementary Figure S3: The effect of Crenolanib on the growth of BCPAP-Empty and BCPAP-PDGFRα cells following a 5-day treatment with the indicated concentrations as measured by MTS assay. Cell growth inhibition was expressed as a percentage of the untreated control cells (means ± SEM, n = 5).

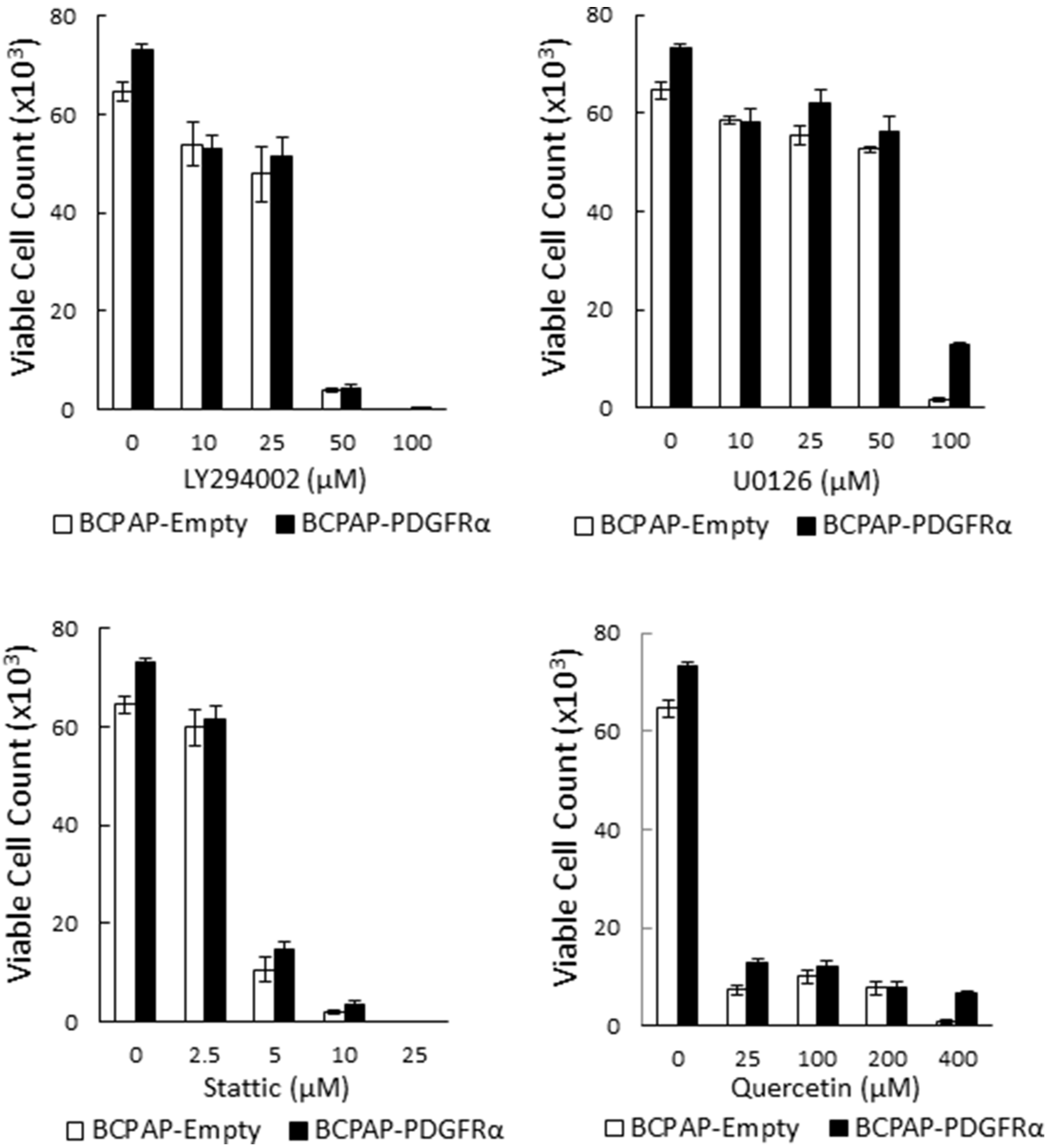

Supplementary Figure S4: PDGFRα expression has no significant effect on the proliferative abilities of PTC cells in the absence or presence of the inhibitors. BCPAP cells lacking or expressing the α-receptor were treated with the indicated doses of inhibitors for 24 h and trypan blue exclusion assay was performed. Results are expressed as total number of viable cells (means ± SEM, n = 6).

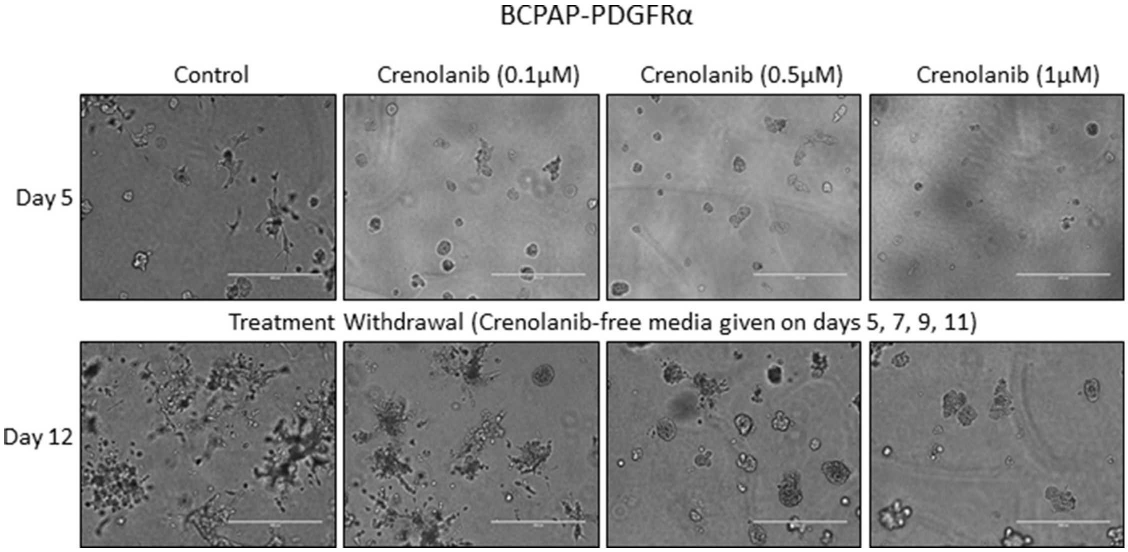

Supplementary Figure S5: The abrogative effect observed with Crenolanib was assessed for its reversibility over a range of concentrations. PDGFRα-expressing cells were grown in 3D Matrigel culture with the concentration of Crenolanib indicated. Treatment was withdrawn after 5 days of propagation by feeding the cells with Crenolanib-free media on days 5, 7, 9 and 11. Phase contrast images on days 5 and 12. Scale bars are 400 μm.

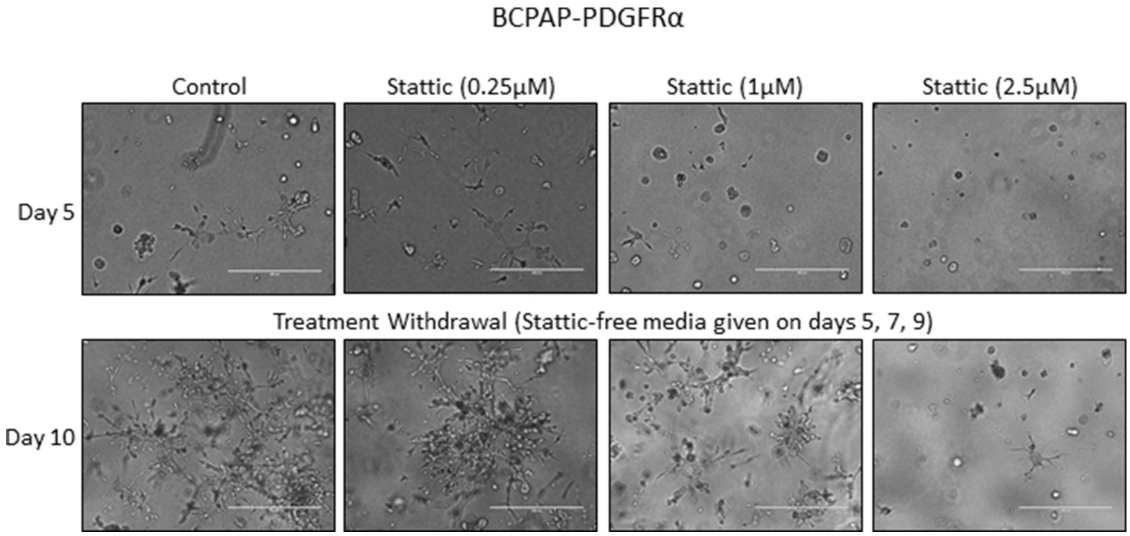

Supplementary Figure S6: The strong inhibitory effect of Stattic on the PDGFRα-induced 3D morphology was assessed for its reversibility over a concentration range (0.25 µM, 1 µM, and 2.5 µM). Treatment of PDGFRα expressing cells with Stattic was discontinued after 5 days, and cells were allowed to grow for another 5 days. Phase contrast images on days 5 and 10. Scale bars are 400 µm.

Supplementary Table S1: List of antibodies and dilutions used for western blotting

Protein	Supplier/Catalogue Number	Dilution
Phospho-Akt	Cell Signalling (4051)	1:1000
Akt	Cell Signalling (9272)	1:2000
N-Cadherin	Cell Signalling (4061)	1:1000
Phospho-Erk	Cell Signalling (9106)	1:1000
Erk	Santa Cruz (sc-94)	1:2000
Phospho-GSK3β (S9)	Cell Signalling (9336)	1:1000
Phospho-PDGFRα (Y754)	Cell Signalling (2992)	1:1000
PDGFRα	Cell Signalling (5241)	1:1000
Phospho-PDGFRβ (Y751)	Cell Signalling (3166)	1:1000
PDGFRβ	Santa-Cruz (sc-80991)	1:1000
Phospho-PDGFRα/β (Y849/ Y857)	Cell Signalling (3166)	1:1000
Slug	Cell Signalling (9585)	1:1000
Snail	Cell Signalling (3879)	1:1000
Phospho-STAT3	Cell Signalling (9131)	1:1000
STAT3	Cell Signalling (9139)	1:1000
Tubulin	Sigma Aldrich (T6557)	1:10000
Twist-1	Santa Cruz (sc-81417)	1:250
Vimentin	Cell Signalling (5741)	1:1000
